# The disease-subject as a subject of literature

**DOI:** 10.1186/1747-5341-2-10

**Published:** 2007-06-29

**Authors:** Andrea R Kottow, Michael H Kottow

**Affiliations:** 1 Universitat zu Köln, Philosophische Fakultät, Romanisches Seminar, Albertus-Magnus-Platz, 50923, Köln; 2 Universidad Diego Portales, Instituto de Humanidades, Vergara 210, Santiago, Chile; 3 Escuela de Salud Pública, Facultad de Medicina, Universidad de Chile, Independencia 939, Santiago, Chile

## Abstract

Based on the distinction between living body and lived body, we describe the disease-subject as representing the impact of disease on the existential life-project of the subject. Traditionally, an individual's subjectivity experiences disorders of the body and describes ensuing pain, discomfort and unpleasantness. The idea of a disease-subject goes further, representing the lived body suffering existential disruption and the possible limitations that disease most probably will impose. In this limit situation, the disease-subject will have to elaborate a new life-story, a new character or way-of-being-in-the-world, it will become a different subject.

Health care professionals need to realize that patients are not mere observers of their body, for they are immersed in a reassesment of values, relationships, priorities, perhaps even life-plans. Becoming acquainted with literature's capacity to create characters, modify narratives and depict life-stories in crisis, might sharpen physicians' hermeneutic acumen and make them more receptive to the quandaries of disease-subjects facing major medical and existential decisions in the wake of disruptive disease.

## Background

Numerous papers have linked medicine and literature, recognizing that medical practice contains textual elements that might be better understood by resorting to the hermeneutical skills in which literature is especially proficient [1]. Scholars do not agree, though, what the text might be, candidates including the patient's narrative, his body signs, or the process of transforming patient histories into medical language [2]. Perusal of literary texts has also been recommended to train medical students' sensitivity by reading testimonial documents, and to become familiar with the features of clinical encounters of the past [3]. There is hardly any need to reinforce the well established intertwinement of literature and medicine; instead, the present text will focus upon the very specific moment when major disease alienates the subject from its lived body and requires a reappraisal of this relationship. We believe that the subject not only experiences disease, it may also be profoundly modified when confronted with its altered body. As physicians become familiar with the subjective aspects of disease, they might enhance theirs skills in understanding a subject that is now a diseased-subject as it tries to cope with the diseased body. Disease and its sequels redimension the limits and possibilities of the body and, as the subject becomes aware of these modified boundaries, it develops into a disease-subject in search of an narrative adapted to the new circumstances. The point of inflection where the lived body becomes a lived body-cum-disease, constitutes a biographical disruption between the traditional subjective experience of disease, and a modified subject, the disease-subject, whose different mode of being-in-the world requires a new narrative. Frequently depicting the fragmentation of life narratives, literature seems an invaluable tool for understanding the existential reorientation that major disease and its sequels may demand.

The subject is to be distinguished from the concept of person, which is agency related, and from the emphasis on identity over time in the sense of "consistency and continuity" characteristic of the self [4]. For the present discussion, two features appear to be necessary and sufficient to describe the subject: privileged access to internal processes that can only be perceived by the individual who experiences them; and expressivity in terms of the capability of presenting one's inner experiences to the world. For the physician to grasp his patient as a whole person, he must rely on the subject's testimony.

## Medicine and the subject

Viktor v. Weizsäcker wrote profusely in favor of a psychosomatic view of medicine, insisting that human beings, both in health and in disease, constitute a whole where the soul impinges on the body and the body influences the soul. In exploring only the organism, medicine is neglecting essential components of existence and disease. Body and soul are not a unit, but they also are not a duality of substances. "Body and soul *are not *a unit, but they *influence *each other. One detects a tendency to separation, as well as one of unification." [[8]: p.87 italics in the original] Rather, they cohabitate in intimate relationship. Calling upon psychoanalysis, Weizsäcker proposed to explore subjectivity with Freudian concepts and techniques, being convinced that disease has a meaning in terms of a "materialization of conflict" [Ibid]. Fully recognizing the subject, he nevertheless preferred to explore rather than to listen, which may explain why his views paved the way to many moral aspects of medicine, but were rapidly superseded by bioethics [9]. Weizsäcker's position is ambiguous and perhaps noncontributory, but is here mentioned because it does occupy a place in the history of subjectivity and disease, even though current thought on this subject owes more to Merleau-Ponty and his followers.

## The living body and the lived body

It was Merleau-Ponty who first suggested that the human organism must be understood as a body, – an "inborn complex" – and, in addition, as "the vehicle to being-in-the-world" [5]. The body as "Körper" is a "morphologically determined and functionally regulated substrate", whereas the body as "Leib" is a permanently changing structure "determined by given circumstances and their significance", a "personal and wordly phenomenon" [6]. These concepts have gained their place in the biomedical disciplines as the living body – a mechanism in the Cartesian way – and the lived body which is an entity oriented by intentionality and the "way in which the world comes to be."[7] The living body is amenable to scrutiny, causal explanation and therapeutic repair, whereas the lived body, coming from within as an intentional way of being-in-the-world, pertains to subjectivity and can only be known to others if it expresses itself. The lived body is the subjective way of understanding the possibilities and limitations of inserting the living body in the world. The subject – lived body – instructs the living body to act, the living body informs the lived body about what it actually can do, for our life-project is steeped in obstacles and possibilities. This dialogue remains recondite unless the subject exposes it by a variety of means, of which language is the richest in symbolic possibilities.

## The subject of disease

Medical practice is becoming consistently aware of the patient as a subject. Scientifically oriented medicine, inaugurated in the XVIII century with the anatomical gaze described by Foucault [10], flourished in the next 200 years, the patient being seen as a mechanism in need of repair, the dead body of pathology teaching what ailed the living body. Not till well into the 20^th ^century is the patient increasingly expected to contribute to the clinical encounter with a rendering of his experiences of disease. Thus, clinical charts distinguish symptoms – subjective experience of disease – from signs – objective clinical findings – and, although historically and semantically incorrect, the distinction is in current use and serves to emphasize the patient's contribution to the understanding and completeness of the clinical encounter. Doctors continue to see the patient as a hopefully reliable bearer of clinically relevant information provided by his privileged subjective access to her living body: "Patients should not lie to their doctor about their medical history, social history, symptoms, and financial arrangements."[11] Of course they shouldn't, but that isn't asking enough of the patient's narrative nor does it concede the subject much more than lip-service importance.

Patients are individuals whose personal being-in-the-world needs to be known for the sake of a pertinent and more complete healing process [12]: "Thus the hermeneutics of medicine will exhibit a normative structure; it will aim to understand with a view to bringing about a certain goal, a goal it regards as morally praiseworthy: let us understand in order to heal, for healing is a good thing."[13,14]

The medical humanities have helped physicians accept that their exploration of the diseased body becomes richer and more accurate by attending to the privileged information only the patient can render, and much effort is being spent in making the medical reading of patient's narration as rewarding as possible. The self is supposed to know its body and detect its dis-orders; the privileged access of subjectivity is expected to deliver valuable clinical information to the health-care professional. The additional data thus gained is of clinical value, but limited in scope. The underlying assumption is that living body and lived body are cast in an atuned and invariable relationship that is undisturbed even by major events like disease. The subject is a stable and reliable witness of the living body's condition in health and disease.

## The diseased subject

A second category to be considered is the diseased subject, where the malady lies not, or not only, in the body, but also in subjectivity. In autoimmune diseases, the patient's experience of his subjectivity may suffer strong tensions and pathological alterations requiring major adjustments: "What if difference is the embodied substrate of subjectivity rather than sameness or one-ness?... Could we learn to live otherwise?" These are questions asked by a perceptive patient whose autoimmune disease threatens his body as well as his subjective integrity [15]. Or consider subjectivity when directly attacked by such a disease as depression, which both gnaws at the fabric of the lived body and issues paralyzing orders to the living body. Mental diseases, at least in their initial phases, cause a profound sensation of menace to the subjective world and disquietening signs of disintegration of the self.

## The disease-subject

The disease-subject is distinct from the subject of disease – the supposedly undaunted observer of its bodily disorder -, and from the diseased subject, where both the lived and the living body crack under a morbid stress. Falling prey to disease, the subject is confronted with threats – to his work, his interests, his life plan, and isolation – from social relations, friends, perhaps even family members. A new coactivity between lived and living body must ensue, the diseased body setting functional limits and suffering disabilities that the lived body must learn to live with. If " [E]mbodiment is defined as being specifically concerned with the lived experience of one's own body" [16], major morbid disruptions of the body will function as limit-situations leading to a profoundly modified embodiment. The experiential substrate is no longer the familiar body, but a new, as yet unpredictable diseased body; consequently, the disease-subject is no longer the subject it was.

The patient's rendering of his condition is not that of a subjectivity describing its body *cum *disease, but the groping search for life coordinates with a body modified by disease and its possible sequelæ. The disease-subject must tell a different story from the one traditionally expected according to which "the patient's story – narration – of the illness is a central part of the meeting, offering the best way to such individualized knowledge."[17] This is necessary but not sufficient. The patient's narrative is no longer only about disease of the living body, as the physician will have it, but about a limit-situation unleashed by disease and requiring a change in life perspectives. Consequently, the patient is not so much interested in the technical details of therapy, but in the ways different medical alternatives or omissions will palliate the damage done to his life project. A schism of meaning may ensue inasmuch as the health-care professional does not percieve the existential quandaries implicit in the patient's narrative.

Shifting the pertinent narrative from the subject of disease – the witness of bodily dysfunctions – to the disease-subject – the witness of existential disruption -, has a profound influence on the way the patient's narrative evolves and is to be understood. Disease is not merely observed dysfunction, it is also anguish in view of the unknown, vulnerability in the wake of permanent sequelæ, perhaps the shudder of death's proximity. All these components dislodge the familiar relationsip of the subject with its living body, create the disease-subject that is witnessing a human being whose life-world is visibly collapsing and where possible rearrangements and new existential pathways will be gropingly searched for.

## In search of a narrative

The intertwinement of literature and medicine has been prolific and variegated, spanning from Thomas Mann's *The Magic Mountain *to the very perceptive narrative and philosophical writings of K. Toombs, suffering from multiple sclerosis [18]. There are many autobiographical reports by patients suffering from cancer, depression, polio, as well as journals devoted to literature and medicine where the subjectivity of disease is often discussed. What we are trying to stress is the inflection point where a person is thrown out of existential balance by disease and must recompose her life project in the wake of modifications and losses of her bodily abilities and sensibilities. This disruption may not be sensed by the health professional, who treats the patient with the idea of restoring her to the previous state. And it is precisely at this inflection point, where character transformations will occur, that literature may have a sensitizing function, since describing characters and having the narrative skill to show how events modify them is a major achievement of narrative literature. *Pars pro toto*, the following quote is taken from a paper that gives a comprehensive overview on the subject and offers a vast reference list [19]: "Literature's power lies in its ability to call up and articulate feelings and evoke vicarious experience."

The humanities in general, and literature in particular, have been most sensitive to the fact that scientific description of disease does not include its subjective experience. Medicine is a social practice, assisted by science but in no way identical to it. Literature and art create symbolic spaces where disease and health, notions of being ill or feeling healthy, suffering and the intents of healing, are all represented and related to other cultural notions [20]. In order to understand the patient in the objective and subjective dimensions of his diseased being, philosophy and literature have been called upon to shape sympathy, develop empathy, and understand meaning, as well as to enrich insight and vocabulary [21,22]. One may be disinclined to search for meaning in disease itself, but there is no question that people suffering major morbid episodes will have to reorganize their lives and search for new existential meaning for their future.

As the disease-subject comes to terms with its altered and burdened body, it will resort to values and meanings, with which literature is familiar, thus helping to pave the way for physicians' efforts to understand their patients. Perception of the disease-subject becomes all the more important when considering the undeterminateness and limitations of medicine. Their discipline being fallible, physicians tend to improve their practical skills by asking the patient *what *he feels, still a far cry from inquiring *how *he feels about his derangement.

Physicians must accept that major disease may deeply and definitely modify the subject's perception of his body, and that they need special skills to read this new disease-subject. It is our contention that medicine, especially in its more pragmatic forms of practice, does not provide these skills, whereas fictional literature has a long tradition in the creation of subjects, notably in those texts where this creation is part of the narrative.

## Creation and modification of character in literature

Literature's devotion to life crisis is nowhere better depicted than in the opening sentence of F. Kafka's long story *Metamorphosis *(*Die Verwandlung*, 1915): "When George Samsa woke up one morning after a night of restless dreams, he found himself in bed, transformed into a ghastly creature."[23] Ricoeur believes that " [L]iterary narrations and life stories, far from being naturally exclusive, are complementary, despite, or even because of, their contrast." [24] Real life must find its way into a story, but the coherence of this story is "threatened by the disruptive effects of the unforeseeable events that punctuate it (encounters, accidents, etc.)" [25]. The narrative identity is put into extreme tension when self-constancy suffers irreparable damage. In disease, the person *qua *narrating subject looses the core of his plot as his life-world changes and he becomes aware of limitations, disabilities and threats emerging from disease, but also from possible therapeutic efforts.

We have selected two literary pieces that fictionally depict how character can be formed through the influence of a disease situation. By becoming familiar with such literary creations, physicians might better understand the process of character remodelling that a severely diseased person goes through when subjectivity must modify the relationship to its damaged body.

## Literary creation of blindness

Max Frisch's novel *The Wilderness of Mirrors *(*Mein Name sei Gantenbein*, 1964) is a prime example of the literary creation of character within a character-building narrative [26]. Frisch unfolds his character Gantenbein who, having considered a number of possibilities, reinvents himself as a blind man: story-teller and created character become fused. Somehow the novel overturns general ideas about illness, for it is Gantenbein who selects blindness as a specific way of being-in-the-world and of relating to people around him. So blindness is not something that befalls Gantenbein but, to the contrary, it is a construction chosen by the character and acted out in a plausible and consequential way. Gantenbein has to face himself as a person with the limitations he associates with blindness. Acting like a handicapped human being, he has to deal with compassion other people feel for him. Through the impressively vivid narration of the novel, the main character defines himself in terms of a disease he does not have and a disability he portrays without suffering it. Disease becomes a special way of self-reflection, a specific form of being-in-the-world, of relating to reality and to others. Disease in this novel is being construed in a twofold sense: not only is it a literary work – a fictional construction – that represents a blind protagonist, but his blindness is created through narration and discourse by the same protagonist. The novel is a fascinating literary experiment, and much can be learned from the fictional rendering of diseased or handicapped individuals. The clinical element is missing in the novel, so is the suffering, for this is an exercise in character composition and the rendering of existential collapse due to severe disability.

## The case of the missing thumb

A recently published philosophical paper invites the reader to observe his left thumb, its anatomical features reflecting important aspects of his biography. Intentional acts will have eventually shaped the deformations and scars peculiar to this person's thumb, thus illustrating a relationship between the lived body of intentions and the living hand of actions. Intentionality is the hinge between the lived and the living body. Should the thumb be traumatically severed, the affected person will "have to recalculate and relearn [his] actions before they can become fluent and unconscious" [27]. Sudden thumblessness is an element of the living body which may profoundly alter the intentionally of the lived body. A new life-plan may be called for if, for example, a violinist's career is thwarted. The missing thumb becomes a clinical case as medicine is called upon to assist in eliminating the "disturbances and obstacles to action" created by the absent left thumb. But, since the thumb is irretrievably lost, it may be expected that certain actions will become impossible, and medical care must help the patient readjust her possible intentionalities to a now thumbless living body. As the thumb goes, the lived body becomes a disease-subject faced with the necessity of creating a new life-story. Nine fingers tell a different story than ten do, and it is not only a clinical but a life-world story.

## Conclusion

Literature's character-building narratives should assist the physician in understanding the process the disease-subject is going through, and help him move beyond the required technical chores in order to help the patient take decisions from the vantage point of his disease-subject condition. Of course, it is tempting to see in literature a possible scaffold for the patient's new identity formation, but we believe this expectation should be resisted, lest literary narrative be overextended way beyond the possibilities of its hermeneutical insights.

Disease-subjects tell the story of disruption, so we need to find the most adequate technique for perceiving disrupted narratives that tell us how the subject faces a limit-situation and is modified by such dramatic events. At first glance, the need to understand the narrative shift suffered by the subject when becoming a disease-subject might seem restricted to only certain instances of medical practice. In fact, minor derangements do not qualify, for they are not disruptive. Beyond trivial disorders, such readjustments are always necessary after a morbid episode, for medicine rarely heals and restitutes *ad integrum*. After major disease, people will have to modify their adaptive preferences and possibilities [28]. K. Goldstein is very precise about this: "Recovery is a newly achieved state of ordered functioning that operates in the direction of a new individual norm."[29]

The need for existential readjustment is all the more obvious when disease becomes chronic and sequelæ set in. Those who encounter individuals afflicted with chronic diseases will have observed how they unfold a narrative that has coherently incorporated pathography into biography, trascending the acute and turbulent phase of the disease-subject. In the recreated life-story, the lived body has learned to perceive and understand the possibilities and limitations of the injured and handicapped living body. The vast literature on disability is a poignant reminder that hermeneutical skills are still insufficiently developed in the different phases of lived body and living body adjustments to chronic disease [30]. Literature is proficient in creating characters and narrating the vagaries and crises they undergo, offering a hermeneutical blueprint that might help the physician understand that the subjectivity of his patients is not a solid constant, but rather a fragile construct that is remodeled by major life events, of which disease is one of the most probable to occur.

The clinical encounter will invariably require a measure of trust and trustworthiness, in order to remain true to its fiduciary essence [31,32]. Trust is based on deciphering the existential quandaries that burden the patient, who renders his narrative in the expectation that the physician may be capable of trascending mere organic concerns. Understanding the disease-subject's narrative is all the more important if alternative therapeutic courses with different but profound and lasting effects on the patient's quality of life must be contemplated, for the choice will be influenced by the disease-subject and will contribute to the life-story evolving under the new life circumstances.

If medicine is to remain true to its humane spirit, physicians must learn to read the text of disease-subjects, in order to better understand and assist patients who are reassessing and reconstructing their life-world by modifying the intertwinement between lived and living body.

## Authors' contributions

ARK prepared the analysis of the literary texts.

MHK wrote the sections on the theoretical background of the lived and the living body and drafted the manuscript.

All authors read and approved the final manuscript.
